# ISG20L2 suppresses bortezomib antimyeloma activity by attenuating bortezomib binding to PSMB5

**DOI:** 10.1172/jci.insight.157081

**Published:** 2022-10-10

**Authors:** Yan Yang, Yuhan Gao, Jingcao Huang, Zhuang Yang, Hongmei Luo, Fangfang Wang, Juan Xu, Yushan Cui, Hong Ding, Zhimei Lin, Xinyu Zhai, Ying Qu, Li Zhang, Ting Liu, Lingqun Ye, Ting Niu, Yuhuan Zheng

**Affiliations:** 1 Department of Hematology, Institute of Hematology, West China Hospital/State Key Laboratory of Biotherapy and Cancer Center and; 2 State Key Laboratory of Biotherapy and Cancer Center, Sichuan University, Chengdu, China.; 3 Department of Hematology, The Affiliated Hospital of Chengdu University, Chengdu, China.; 4 Center for Translational Research in Hematologic Malignancies, Houston Methodist Cancer Center/Houston Methodist Research Institute, Houston Methodist, Houston, Texas, USA.

**Keywords:** Hematology, Drug therapy

## Abstract

The proteasome inhibitors (PIs) bortezomib and carfilzomib, which target proteasome 20S subunit beta 5 (PSMB5) in cells, are widely used in multiple myeloma (MM) treatment. In this study, we demonstrated the role of interferon-stimulated 20 kDa exonuclease-like 2 (*ISG20L2*) in MM PI resistance. Gain- and loss-of-function studies showed that ISG20L2 suppressed MM cell sensitivity to PIs in vitro and in vivo. Patients with *ISG20L2*^lo^ MM had a better response to PIs and a longer overall survival than patients with *ISG20L2*^hi^ MM. Biotinylated bortezomib pull-down assays showed that ISG20L2 competed with PSMB5 in binding to bortezomib. The surface plasmon resonance assay confirmed the direct binding of bortezomib to ISG20L2. In *ISG20L2*^hi^ MM cells, ISG20L2 attenuated the binding of bortezomib to PSMB5, resulting in lower inhibition of proteasome activity and therefore less bortezomib-induced cell death. Overall, we identified a potentially novel mechanism by which ISG20L2 conferred bortezomib resistance on MM. The expression of *ISG20L2* correlated with MM PI responses and patient treatment outcomes.

## Graphical abstract

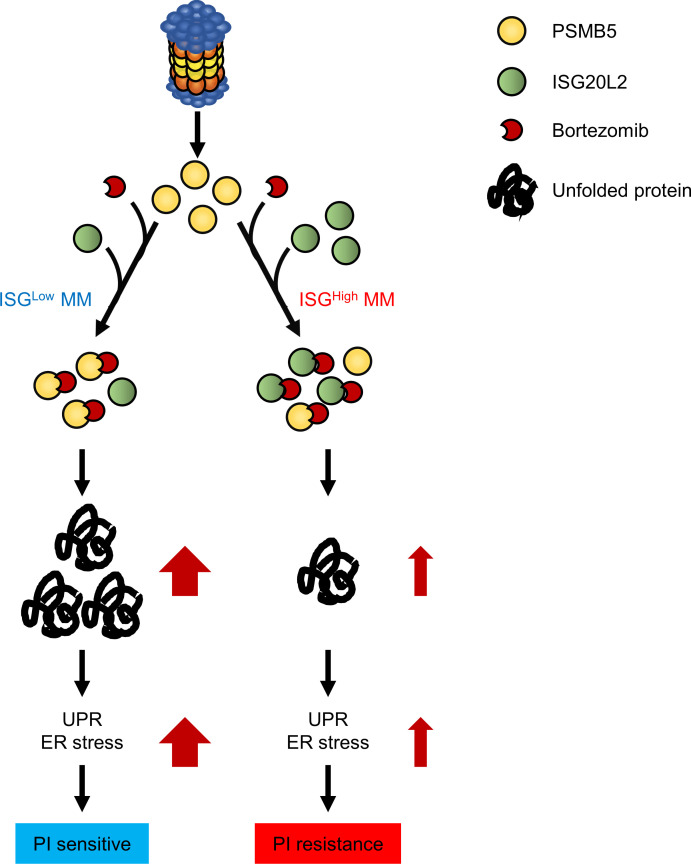

## Introduction

Multiple myeloma (MM) is a refractory hematological cancer characterized by monoclonal plasma cell accumulation in bone marrow (1). Over the past decades, the use of proteasome inhibitors (PIs) has significantly improved the prognosis of patients with MM. Bortezomib (BTZ), also known as Velcade or PS-341, was the first PI approved by the US Food and Drug Administration for treating MM. Due to its effectiveness and good tolerability, BTZ has become central to various MM treatment regimens (2). However, BTZ resistance is common in patients with relapsed and refractory MM, and the underlying mechanisms are still not fully understood.

MM with chromosome 1q amplification (+1q) is considered high risk and is likely to respond poorly to BTZ (3–5). We identified interferon-stimulated 20 kDa exonuclease-like 2 (*ISG20L2*) in our study of prognostic genes in 1q. *ISG20L2* is in the 1q23 region of chromosome 1. Upregulation of *ISG20L2* was previously found in patients with 1q-amplified MM (6). Another report showed that *ISG20L2* was among the top 34 genes that could predict a high risk of progression from smoldering MM to symptomatic MM (7). Nevertheless, the roles of ISG20L2 in MM progression remain obscure. ISG20L2 is known as an exoribonuclease (8). Microarray-based analysis showed that *ISG20L2* expression correlated with overall survival (OS) in MM. In this study, we show that ISG20L2 confers BTZ resistance on MM cells via a potentially novel mechanism.

## Results

### Identification of chromosome 1q prognostic gene expression in MM.

**Figure 1:**
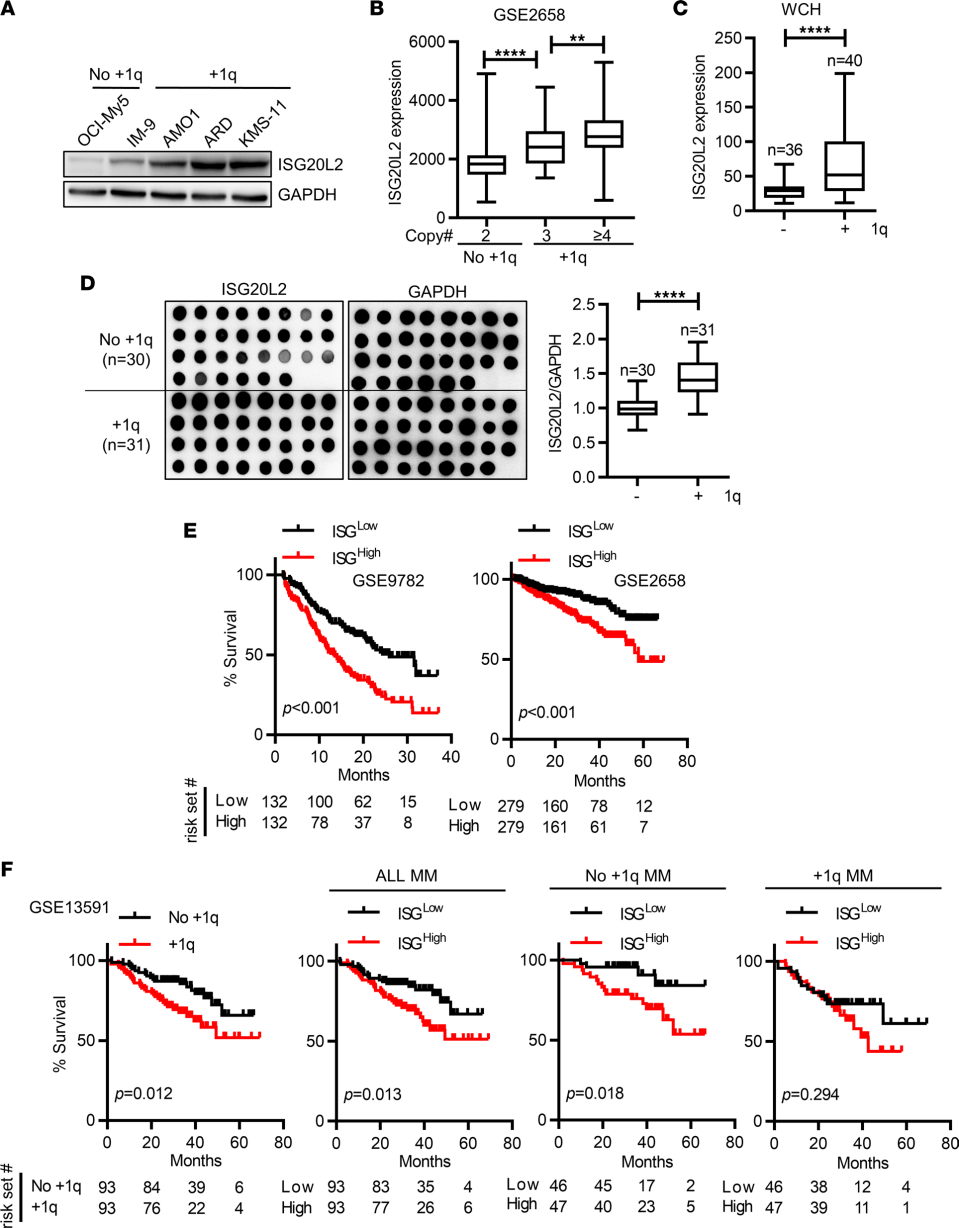
Identification of ISG20L2 as an overexpressed gene in +1q myeloma. (**A**) Western blot showing the expression of ISG20L2 in 5 human MM cell lines (OCI-My5, IM-9, AMO1, ARD, KMS-11). (**B**) In the MM patient gene expression data set GEO GSE2658, the expression of *ISG20L2* in primary MM cells increased with the copy number of 1q21 (*n* = 248; patients with 2 copies = 134, patients with 3 copies = 70, patients with 4 or more copies = 44). One-way ANOVA with a post hoc least significant difference (LSD) *t* test was performed. *P* values: **P ≤ 0.01; ****P ≤ 0.0001. (**C**) Real-time quantitative PCR (RT-qPCR) results showed *ISG20L2* expression in primary MM cells of 76 patients diagnosed at West China Hospital (WCH). The 1q amplification status of patients was determined by FISH assay. Student’s *t* test was performed. ****P ≤ 0.0001. (**D**) Dot blot assay of ISG20L2 in the 61 patients with MM mentioned in **C** (left blots). The dot intensities of ISG20L2 relative to GAPDH were calculated (right column). Student’s *t* test was performed. ****P ≤ 0.0001. Box plots show the interquartile range (box), median (line), and minimum and maximum (whiskers). (**E**) In MM patient gene expression data sets GSE9782 (left, *n* = 264) and GSE2658 (right, *n* = 559), Kaplan-Meier curves showed the correlation between *ISG20L2* expression and overall survival (OS) in patients with MM. *P* values were calculated by Mantel-Cox test. (**F**) In the GSE13591 data set (*n* = 186), Kaplan-Meier curves showed the survival of different MM patient groups. *P* values were calculated by Mantel-Cox test.

MM with +1q results in the amplification of multiple 1q genes (9). Therefore, we included all protein-encoding genes in 1q (1q12–1q44) in our analysis. According to the EnsemblGenomes website (https://ensemblgenomes.org/), a total of 1,984 protein-coding genes were located on 1q (1q12–1q44). Using the NCBI Gene Expression Omnibus (GEO) GSE2658 data set, we selected genes overexpressed in +1q MM in a copy number–dependent manner and further identified prognostic genes. We identified 5 candidate genes, *ILF2*, *VPS72*, *ISG20L2*, *CDC42SE1*, and *TADA1*. In this study, we focused on *ISG20L2* function in MM (Supplemental Figure 1A; supplemental material available online with this article; https://doi.org/10.1172/jci.insight.157081DS1). *ISG20L2* is located in the 1q23 region. In clinical practice, fluorescence in situ hybridization (FISH) assays often use 1q21 probes to identify the amplification of +1q (6). We performed dual-color FISH assays using 1q21 and 1q23 probes, showing that the amplifications of 1q21 and 1q23 were identical in the tested primary MM cells and human MM cell lines (Supplemental Figure 1, B and C). MM cell lines or patients with a high copy number of 1q showed high ISG20L2 expression (Figure 1, A and B). We observed higher ISG20L2 expression at both the mRNA and protein levels in +1q MM patients than in patients without +1q (Figure 1, C and D). Finally, we verified the prognostic value of *ISG20L2* expression in different MM data sets. *ISG20L2*^hi^ MM had inferior OS (Figure 1, E and F). Interestingly, for patients with MM without +1q, *ISG20L2* expression also correlated with MM OS (Figure 1F). For +1q MM, *ISG20L2*^hi^ patients had a worse prognosis but without statistical significance (Figure 1F). This may be attributed to the limited number of +1q patients (*n* = 93) in the data set (GEO GSE13591) or the already high expression of *ISG20L2* in +1q MM. These findings offer proof of the prognostic value of *ISG20L2* in MM. Importantly, previous studies have identified multiple functional 1q genes in myeloma pathogenesis and chemoresistance (6, 7, 10). The correlation of *ISG20L2* expression and other prognostic factors in the +1q cohort has not yet been determined. Our understanding of the roles of *ISG20L2* in MM is still very limited.

### ISG20L2 regulates MM drug sensitivity to PIs.

**Figure 2:**
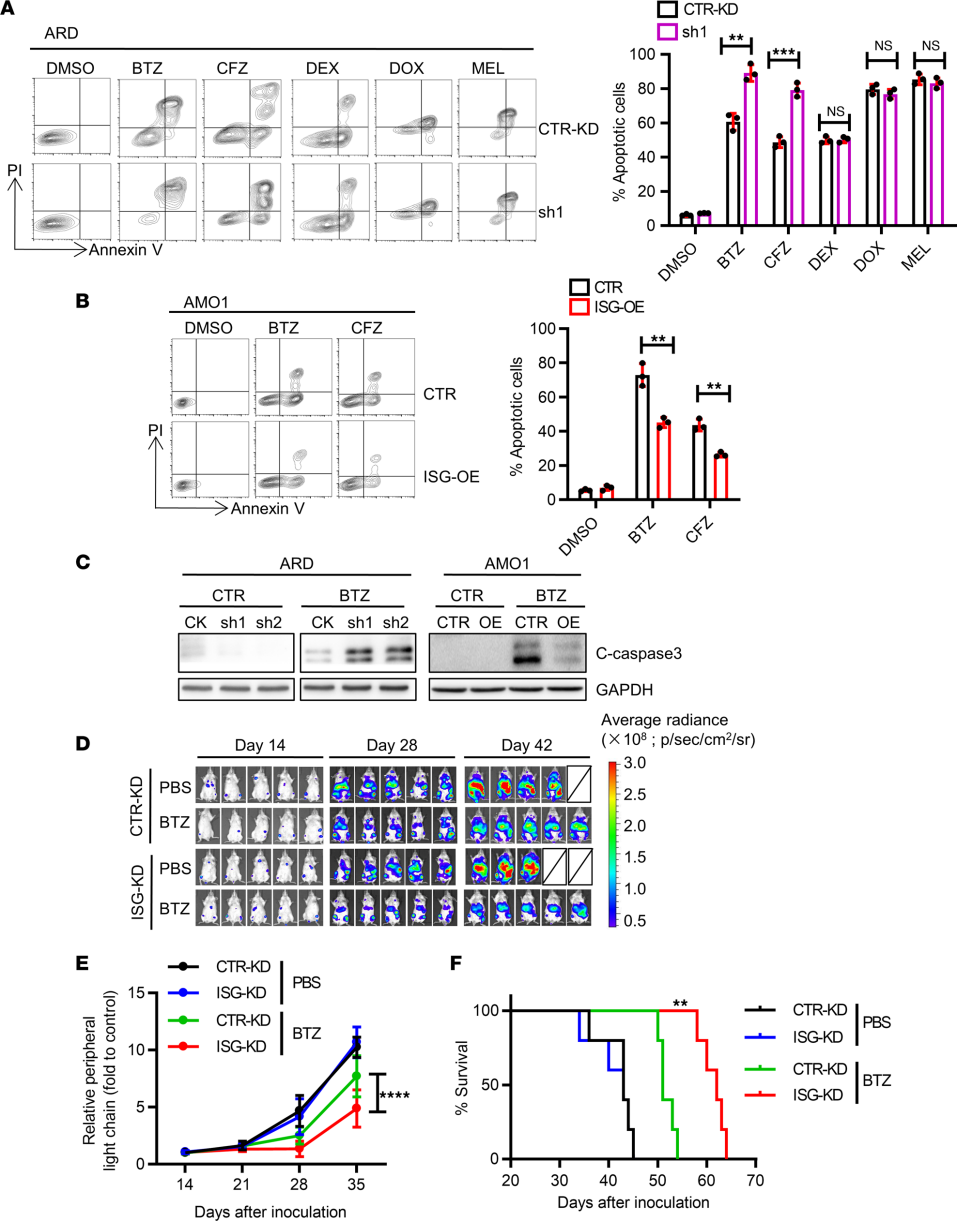
ISG20L2 regulates MM cell sensitivity to PIs. (**A**) CTR-KD and ISG-KD (sh1 used) ARD cells were treated with different drugs for 24 hours. BTZ (5 nM), CFZ (3.5 nM), doxorubicin (DOX, 2 μM), melphalan (MEL, 15 μM), and dexamethasone (DEX, 40 μM) were used. Cell apoptosis was analyzed by flow cytometry with annexin V and propidium iodide (PI) double staining (left), and the percentage of apoptotic cells was quantified (right, *n* = 3). Student’s *t* test was performed. **P ≤ 0.01; ***P ≤ 0.001. (**B**) AMO1 cells infected with control virus (CTR) or ISG-OE were treated with BTZ (5 nM) and CFZ (3.5 nM) for 24 hours. Cell apoptosis was analyzed (left), and the results were quantified (right, *n* = 3). Student’s *t* test was performed. **P ≤ 0.01. (**C**) CTR-KD versus ISG-KD ARD cells and CTR versus ISG-OE AMO1 cells were treated with BTZ (5 nM for 12 hours). Western blot results of cleaved caspase-3 and GAPDH are shown. (**D**) CTR-KD or ISG-KD (sh1 used) ARD cells expressing luciferase were intravenously injected into B-NDG mice to establish a human MM xenograft mouse model. Bioluminescence imaging indicated a correlation between ISG20L2 expression and MM sensitivity to BTZ in vivo (*n* = 5 for each group). (**E**) Monoclonal protein (human κ light chain secreted by ARD cells) levels in mouse peripheral blood were examined by ELISA. Two-way ANOVA with Tukey’s post hoc test was performed. (****P < 0.0001 for BTZ-treated CTR-KD vs. ISG-KD mice.) (**F**) Survival curves of B-NDG mice with different treatments. The Mantel-Cox test was performed. (**P < 0.01 for CTR-KD vs. ISG-KD mice treated with BTZ.)

To examine ISG20L2 function, we generated human MM cell lines with consistent *ISG20L2* knockdown (ISG-KD) and overexpression (ISG-OE) (Supplemental Figure 2A). ISG-KD did not affect cell growth of the ARD line in vitro (Supplemental Figure 2B) but enhanced its sensitivity to BTZ or carfilzomib (CFZ) (Figure 2A). ISG-KD did not significantly change MM sensitivity to DEX (40 μM), DOX (2 μM), or MEL (15 μM) at the concentration we used. Further studies may be required to test the functions of ISG20L2 in MM multidrug resistance. ISG-KD ARD cells were sensitive to a wide dose range (3.125 to 50 nM) of BTZ. When more than 80% of both CTR-KD and ISG-KD cells were killed at 12.5 nM BTZ, BTZ still induced more late apoptotic (annexin V^+^PI^+^) cells in ISG-KD cells (Supplemental Figure 2C). ISG-OE AMO1 cells showed decreased sensitivity to PIs compared with CTR cells (Figure 2B). Apoptotic cleaved caspase-3 fragmentation indicated differences in BTZ-induced cell death in control cells and ISG-KD or ISG-OE cells (Figure 2C). Similar results were also observed in the ISG-KD human MM cell line KMS-11 (Supplemental Figure 2, D–G). To test the above results in vivo, we generated a human MM xenograft mouse model using CTR-KD and ISG-KD ARD cells. Tumor-bearing mice were treated with a low dose of BTZ (0.75 mg/kg, twice weekly for 4 weeks) using PBS as a control. The tumor burden was examined by in vivo luminescence assay (Figure 2D) and circulating monoclonal protein (Figure 2E). Although CTR-KD and ISG-KD MM exhibited similar growth in vivo without drug treatment, ISG-KD MM was more sensitive to BTZ treatment than CTR-KD MM in vivo. ISG-KD MM-bearing mice survived longer than CTR-KD MM-bearing mice after BTZ treatment (Figure 2F).

**Figure 3:**
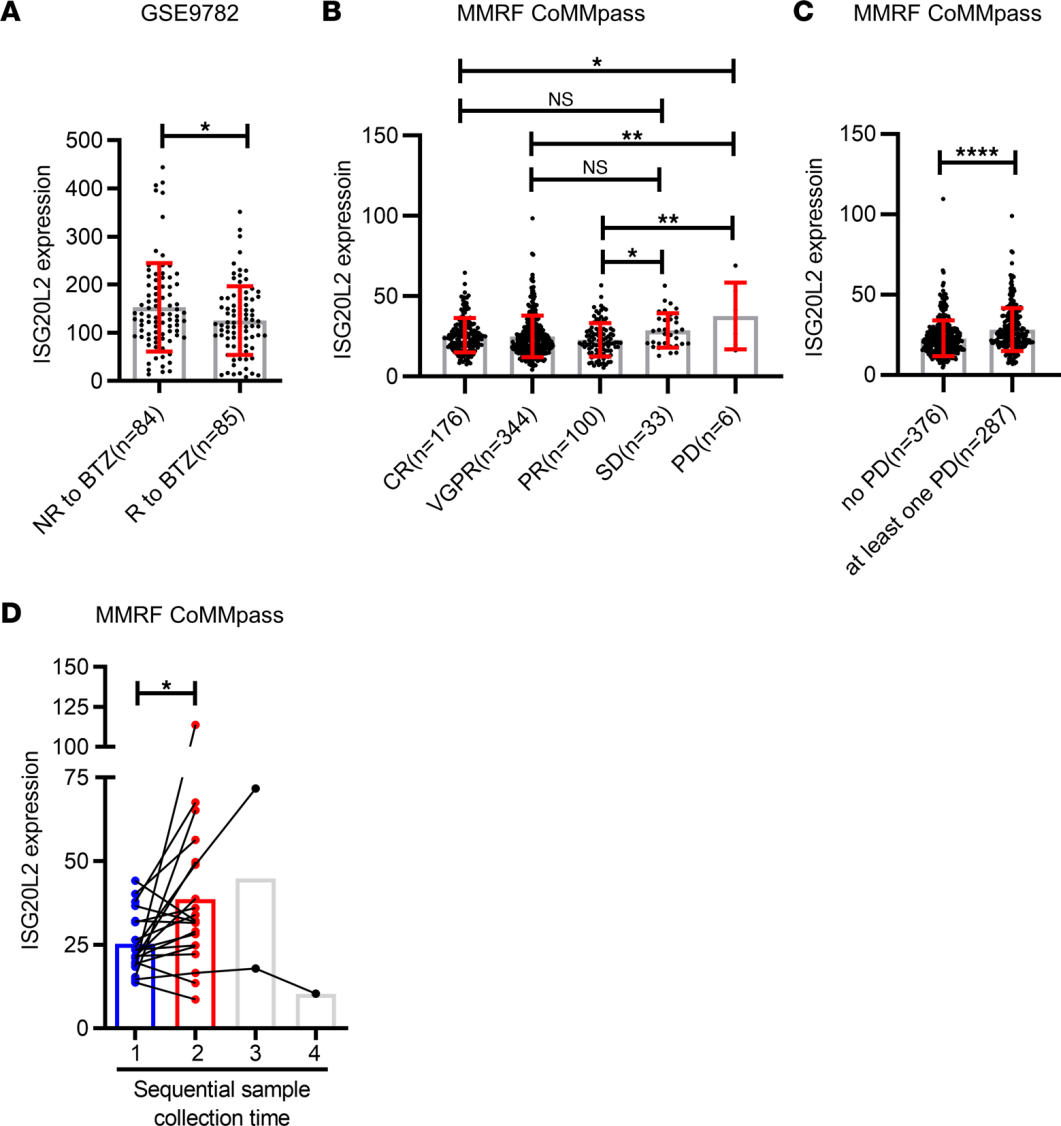
ISG20L2 expression correlates with the response of patients with MM to PIs. (**A**) In data set GSE9782, 169 patients with MM received BTZ treatment. We analyzed *ISG20L2* expression in patients with MM who responded to BTZ treatment (R, *n* = 85, *ISG20L2* expression: 8.009 to 351.6, median value = 118.6) and who did not respond to BTZ treatment (NR, *n* = 84, *ISG20L2* expression: 13.89 to 443.7, median value = 134.5). Student’s *t* test was performed. *P ≤ 0.05. (**B**) In the MMRF CoMMpass data set, analyses of the correlation between ISG20L2 expression and patients’ response status to PIs (BTZ or CFZ) treatment. Patients were stratified based on response degrees after treatment as CR (*n* = 176, *ISG20L2* expression: 7.612 to 64.42, median value = 24.83), VGPR (*n* = 344, *ISG20L2* expression: 4.044 to 98.31, median value = 21.94), PR (*n* = 100, *ISG20L2* expression: 5.433 to 56.73, median value = 21.07), SD (*n* = 33, *ISG20L2* expression: 12.84 to 56.51, median value = 27.83) and PD (*n* = 6, *ISG20L2* expression: 16.14 to 68.98, median value = 33.86). One-way ANOVA with post hoc LSD *t* test was performed. *P ≤ 0.05; **P ≤ 0.01. (**C**) In the MMRF CoMMpass data set, for the patients who received PI treatment, ISG20L2 expression was analyzed in patients without PD (*n* = 376, *ISG20L2* expression: 4.044 to 108.9, median value = 20.88) and in patients with at least 1 PD (*n* = 287, *ISG20L2* expression: 6.284 to 98.31, median value = 25.33). Student’s *t* test was performed. ****P ≤ 0.0001. (**D**) In the MMRF CoMMpass data set, for the patients who received PI-based therapy and died from disease progression, 20 patients had sequential BM gene expression profile data. *ISG20L2* expression increased during the treatment. Student’s *t* test was performed. *P ≤ 0.05.

Gene expression profiling data sets from patients with MM also suggested that *ISG20L2* expression correlated with patients’ response to PIs. In the GSE9782 data set (11), patients in the trial were classified as achieving complete response, partial response, minimal response, no change, or progressive disease. We defined the complete response, partial response, and minimal response groups as “response to BTZ,” while the no change and progressive disease groups were defined as “no response to BTZ.” The patients who did not respond to BTZ had higher *ISG20L2* expression at diagnosis than those who responded to BTZ (Figure 3A). Using the Multiple Myeloma Research Foundation (MMRF) CoMMpass data set (12), we analyzed data from patients who had been treated with PIs (BTZ or CFZ). After PI treatment, patients who had progressive disease (PD) had higher *ISG20L2* expression than those with complete response (CR), very good partial response (VGPR), or partial response (PR). Patients with stable disease (SD) had higher median and mean values of *ISG20L2* expression than the CR, VGPR, and PR groups, but the difference was statistically significant only when compared with the PR group (Figure 3B). Considering the different 1q amplification statuses of patients, for +1q patients, only 1 patient ended up in the PD group, which was not sufficient for statistical analyses. The expression of *ISG20L2* was slightly higher in the SD group than in the CR, VGPR, and PR groups, but the difference was not statistically significant. We found that in patients without +1q, *ISG20L2* expression was higher in the PD group than in the CR, VGPR, and PR groups. No +1q patients with SD had higher *ISG20L2* expression than the CR, VGPR, and PR groups, but the difference was not statistically significant when compared with the CR group (Supplemental Figure 3A). For patients treated with the PI-based regimen, those with no PD had lower *ISG20L2* expression than those with at least 1 PD (Figure 3C). Patients with at least 1 PD had higher *ISG20L2* expression, regardless of their 1q amplification status (Supplemental Figure 3B). Most patients who received PI-based therapy and died from disease progression had *ISG20L2* upregulation during the treatment course (Figure 3D). Of note, the above statistically significant comparison results based on MM patient data were quantitatively modest.

To summarize, we found that ISG20L2 regulated MM PI sensitivity in vitro and in vivo. MM patients with higher *ISG20L2* expression were less sensitive to PIs and had inferior treatment outcomes.

### ISG20L2 competes with the beta 5 subunit of the 20S proteasome complex in BTZ binding.

**Figure 4:**
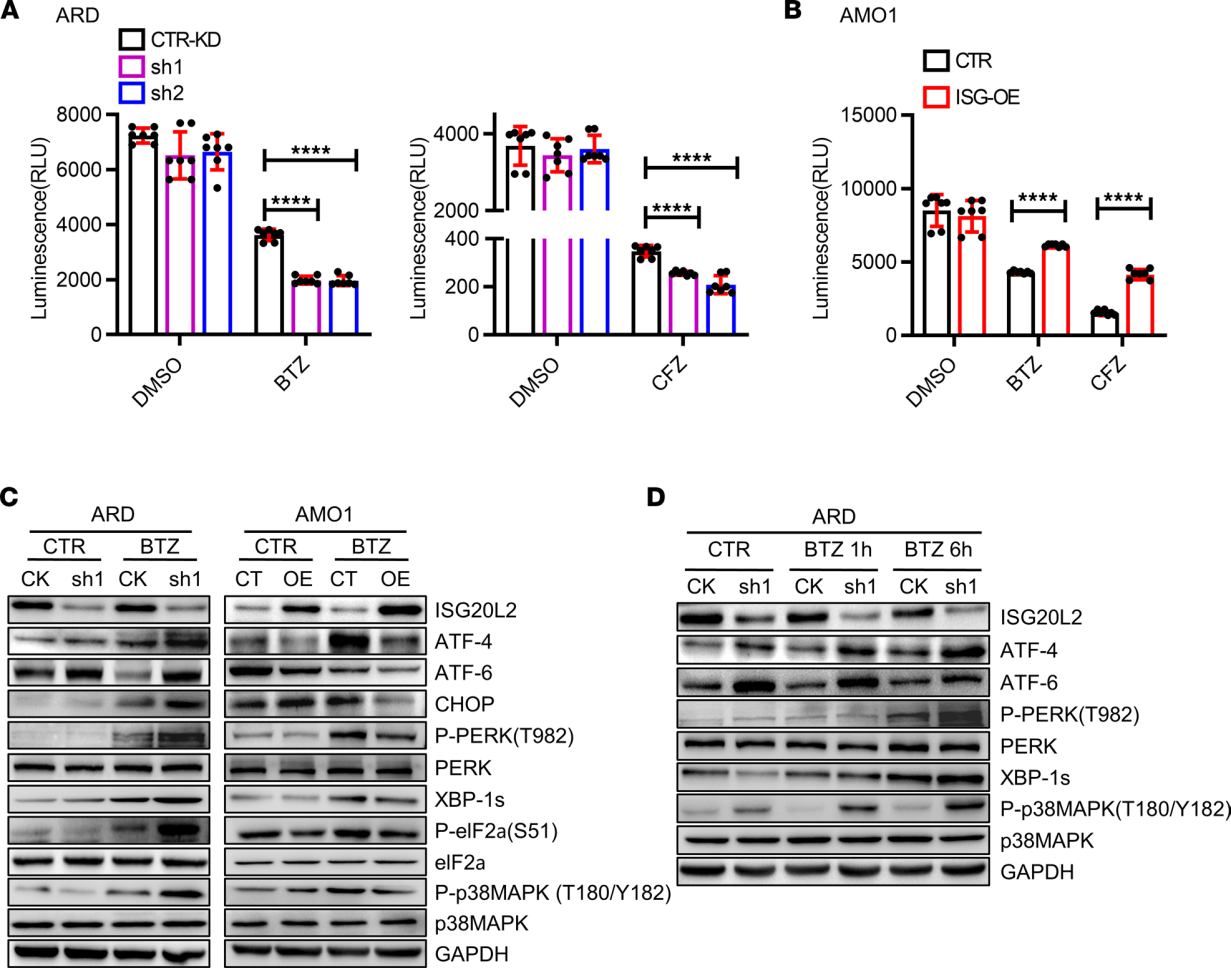
ISG20L2 regulates ubiquitin-proteasome–related cell signaling. (**A**) CTR-KD and ISG-KD ARD cells that were treated with BTZ (5 nM, 1.5 hours, left) and CFZ (3.5 nM, 1.5 hours, right). The solvent DMSO served as a negative CTR. Proteasome activity was determined by measuring the fluorescence (amc) intensity released by the cleavage of the fluorogenic substrate suc-LLVY-amc (*n* = 6). Student’s *t* test was performed. ****P ≤ 0.0001. (**B**) Proteasome activity of CTR and ISG-OE AMO1 cells pretreated with BTZ (5 nM, 1.5 hours) or CFZ (3.5 nM, 1.5 hours) (*n* = 6). Student’s *t* test was performed. ****P ≤ 0.0001. (**C**) CTR-KD versus ISG-KD ARD cells, as well as CTR versus ISG-OE AMO1 MM cells, were treated with BTZ (5 nM, 4 hours). The cell lysates were used for Western blotting to examine UPR pathway activation. The UPR pathway components ATF6, PERK, p-PERK (T982), eIF2α, p-eIF2α (S51), ATF4, CHOP, and XBP-1s were analyzed. p38MAPK and p-p38MAPK (T180/Y182) were also examined. (**D**) Western blot results showed the signaling molecules in UPR and ER stress pathways in CTR-KD versus ISG-KD ARD cells, which were treated with BTZ (5 nM) for 1 hour or 6 hours.

We examined proteasome activity in CTR-KD versus ISG-KD ARD cells. PI exposure resulted in greater proteasome inhibition in ISG-KD cells than in CTR-KD cells (Figure 4A); conversely, less proteasome inhibition was observed in ISG-OE cells (Figure 4B). Unfolded protein response (UPR) signaling is known to mediate PI-induced MM cell death (13, 14). Western blot results showed that BTZ treatment induced upregulation of ATF4, ATF6, CHOP, phosphorylated PERK (p-PERK) (T982), XBP-1s, and p-eIF2a (S51) in ISG-KD ARD cells (Figure 4, C and D). Consistent results were observed in ISG-OE AMO1 cells (Figure 4C).

**Figure 5:**
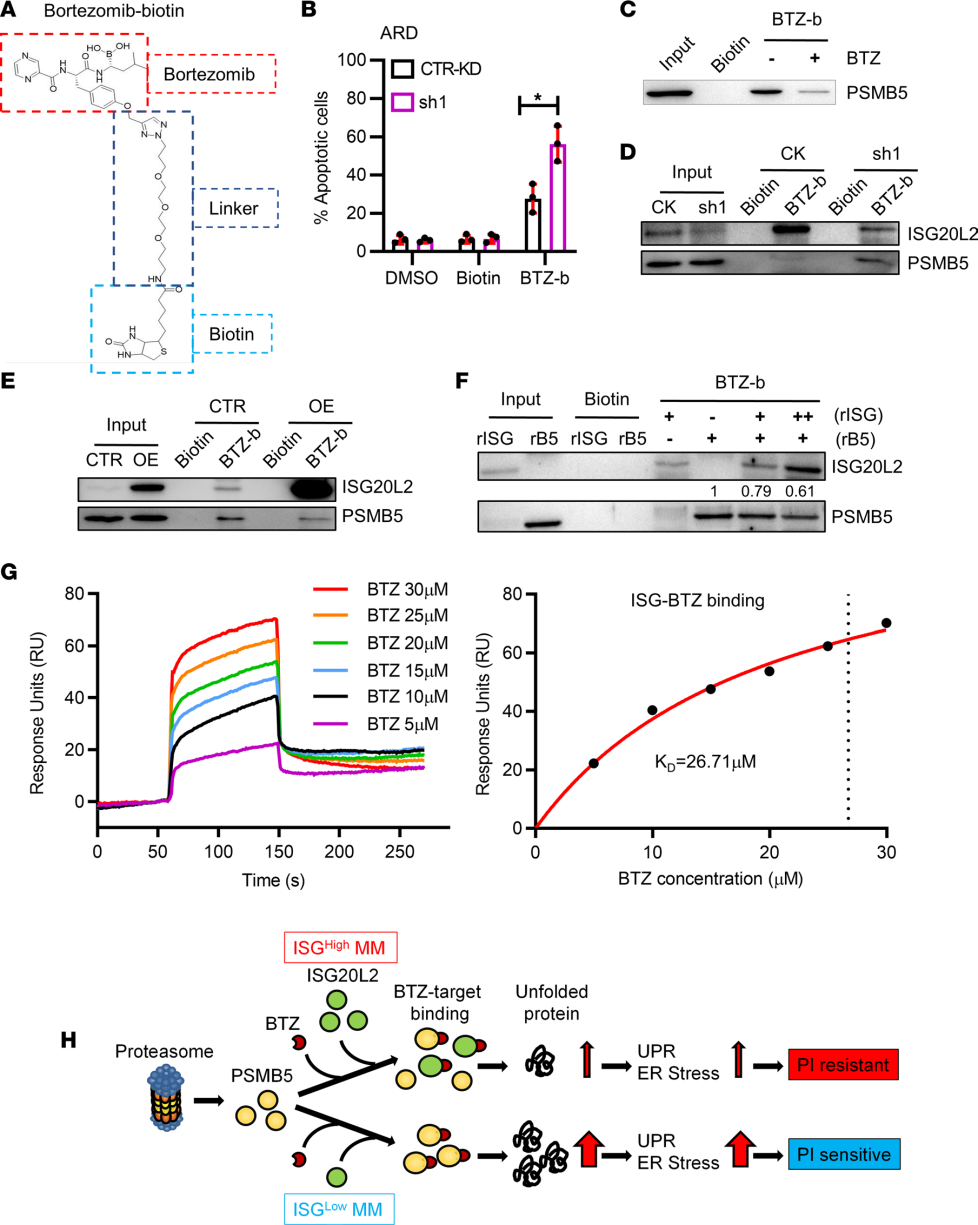
ISG20L2 competes with PSMB5 in BTZ binding. (**A**) Molecular structure of biotinylated BTZ (BTZ-b). (**B**) Flow cytometry–based apoptosis assay to examine the cytotoxicity of BTZ-b in CTR-KD versus ISG-KD (sh1) ARD cells. The cells were treated with BTZ-b (2 μM) for 24 hours. DMSO- or biotin-treated cells served as CTRs (*n* = 3). Student’s *t* test was performed. *P ≤ 0.05. (**C**) BTZ-b pull-down assay using ARD cell lysate in the presence of BTZ to examine BTZ competition with BTZ-b in PSMB5 binding. BTZ was added to the cell lysate to a final concentration of 200 μM and incubated with BTZ-b–coated beads for competition. (**D**) Biotinylated BTZ pull-down assay using CTR-KD and ISG-KD ARD cell lysates. Biotin served as a negative control for pull-down. The pull-down precipitate and the whole-cell lysates (input) were subjected to Western blotting. (**E**) BTZ-b pull-down assay using CTR and ISG-OE AMO1 cell lysates. (**F**) BTZ-b pull-down assay using recombinant ISG20L2 (rISG) and PSMB5 (rB5) proteins. The recombinant protein was dissolved in PBS. The final concentration of rB5 was 0.5 μg/mL (+), while the final concentration of rISG was 0.25 μg/mL (+) or 1.5 μg/mL (++). Using the gray intensity of PSMB5 pull-down by BTZ-b alone as a control, ImageJ software (NIH) was used to determine the levels of PSMB5 pull-down by BTZ-b with rISG competition. Numbers indicated on the lane. (**G**) BTZ binds to ISG20L2 as shown by SPR measurements. Graphs of equilibrium response unit (RU) responses versus compound concentrations were plotted. The estimated *K_D_* is 26.71 μM, as shown by the vertical dotted line. (**H**) Schematic mechanism of ISG20L2-induced MM BTZ resistance.

The beta 5 subunit of the 20S proteasome complex (PSMB5) was identified as a molecular target of BTZ (15). We synthesized biotinylated bortezomib (BTZ-b) (Figure 5A). The compound BTZ-b induced the same pattern of cell death in CTR-KD versus ISG-KD ARD cells as that observed with BTZ treatment (Figure 5B). A competition BTZ-b pull-down assay showed that the addition of BTZ to ARD cell lysate decreased the binding of PSMB5 to BTZ-b (Figure 5C). This result suggested that the BTZ-b analog mimicked BTZ in PSMB5 binding. The BTZ-b pull-down assay using ARD cell lysate showed that both PSMB5 and ISG20L2 could be pulled down by BTZ-b (Figure 5D). More PSMB5 was pulled down in ISG-KD MM than in CTR-KD MM (Figure 5D), while ISG-KD had a mild effect on the expression of the 20S proteasome β subunits (Supplemental Figure 4A). Consistent results were observed in BTZ-b pull-down assays using ISG-OE MM cell lysate (Figure 5E) or PSMB5-KD MM cell lysate (Supplemental Figure 4B). Next, we used recombinant ISG20L2 (rISG) and PSMB5 (rB5) proteins to repeat the pull-down assay (Figure 5F). The addition of rISG decreased the pull-down of PSMB5 by BTZ-b in a dose-dependent manner. To confirm the specific binding of BTZ to ISG20L2, we performed surface plasmon resonance (SPR) assays, confirming that the binding affinity of BTZ to ISG20L2 (*K_D_* = 26.71 μM) was comparable to that of BTZ binding to PSMB5 (*K_D_* = 25.05 μM) (Figure 5G and Supplemental Figure 5A). Additionally, through SPR assays, we confirmed the specific binding of CFZ to ISG20L2, and the affinity between ISG20L2 and CFZ (*K_D_* = 8.712 μM) was similar to that between PSMB5 and CFZ (*K_D_* = 15.31 μM) (Supplemental Figure 5, B and C). Based on our findings, we proposed the mechanism of ISG20L2-induced MM resistance to PIs (Figure 5H). In *ISG20L2*^lo^ MM cells, BTZ mainly bound to PSMB5 and inhibited proteasome activity. This inhibition resulted in a UPR in MM cells and eventual cell death. In contrast, in *ISG20L2*^hi^ MM, substantial amounts of BTZ bound to ISG20L2 instead of the proteasome complex. Therefore, *ISG20L2*^hi^ MM was less sensitive to BTZ. To our knowledge, the ISG20L2 protein structure has yet to be revealed. Crystallographic data showing binding of BTZ with ISG20L2 might provide confirmation of the PI resistance mechanism that we proposed here.

## Discussion

Resistance to PIs, such as BTZ and CFZ, remains a major limitation to the full application of drugs in MM treatment. The proteasome β 5 subunit, encoded by the *PSMB5* gene, is the primary molecular target of BTZ. *PSMB5* gene mutations that affected the structure of the BTZ binding pocket of the PSMB5 protein resulted in impaired BTZ binding and therefore decreased drug sensitivity (16, 17). Since second-generation PI CFZ targeted PSMB5 as well, many MMs with BTZ resistance mutations in PSMB5 also responded poorly to CFZ or ixazomib (18, 19). In addition to mutations, the abundance of the PSMB5 protein also correlated with BTZ resistance. MM with high PSMB5 expression had decreased BTZ sensitivity (20, 21). These findings might indicate that the compound-to-target ratio was also critical to achieve effective proteasome inhibition in BTZ treatment. In our study, we showed that a high level of ISG20L2 prevented BTZ from binding to its anti-MM target PSMB5. Therefore, reduced proteasome inhibition, weaker downstream UPR signaling, and less BTZ-induced cell death were observed.

The function of ISG20L2 in cells is still largely unknown. Previously, Coute et al. reported that ISG20L2 is an exoribonuclease involved in ribosomal subunit biosynthesis (8). Recently, several independent omics analyses identified ISG20L2 as a prognostic marker in different human cancers, including hepatocellular carcinoma (22–24), lung adenocarcinoma (25), and breast cancer (26). Using MM gene expression profiles, we also found that high ISG20L2 expression was associated with inferior MM OS. Furthermore, we addressed the negative impact of ISG20L2 on MM prognosis by showing that ISG20L2 might function as an interference target of BTZ in MM cells. Mounting evidence suggests that PI resistance is mediated by multiple mechanisms. Our findings demonstrated a role for ISG20L2 in MM PI resistance. However, how much ISG20L2 contributes to MM patient resistance to PI-based therapy was not determined in our study.

To our knowledge, PIs are not used in the treatment of hepatocellular cancer, lung cancer, or breast cancer. Therefore, the oncogenic role of ISG20L2 across cancers remains uncertain and requires further investigation.

## Methods

### Patient samples.

Bone marrow aspirations from 76 patients with MM were used in this study. Patient samples were obtained from the tissue bank of the Department of Hematology, West China Hospital, Sichuan University. This study was approved by the Ethical Committee of West China Hospital, Sichuan University (Protocol No. 114). Written informed consent was obtained from the patients or their legal guardians for sample collection and usage.

Bone marrow mononuclear cells were isolated by Ficoll density gradient centrifugation. CD138^+^ cells were sorted by immune magnetic beads (Miltenyi Biotec, catalog 130-051-301) according to the manufacturer’s protocol.

### Myeloma gene expression profile data sets.

The MM gene expression profile (GEP) data sets GSE13591, GSE755, GSE2658, and GSE9782 were downloaded from the NCBI GEO database (https://www.ncbi.nlm.nih.gov/geo). The corresponding clinical information was obtained from the Oncomine database (https://www.oncomine.org). The GEP and clinical information from the MMRF CoMMpass study was downloaded from the MMRF web portal (https://research.themmrf.org).

For the GSE755 data set, CD138^+^ plasma cells were isolated from newly diagnosed MM patients’ (*n* = 173) bone marrow. The GEPs were assessed using the Affymetrix U95Av2 microarray platform. For the GSE2658 data set, CD138^+^ plasma cells were isolated from newly diagnosed MM patients’ (*n* = 559) bone marrow. The patients were subsequently treated with high-dose therapy and stem cell transplantation, referred to as total therapy 2 and total therapy 3. The GEPs were assessed using the Affymetrix U133Plus2.0 microarray platform. For the GSE9782 data set, plasma cells were negatively selected from 264 patients with relapsed MM. Those patients were enrolled in phase II/III clinical trials for BTZ (PS-341). The GEPs were assessed using the Affymetrix U133A/B microarray platform.

### Reagents and antibodies.

BTZ (catalog S1013), CFZ (catalog S2853), MEL (catalog S8266), DEX (catalog S1322), and DOX (catalog S1208) were purchased from Selleck Chemicals. Recombinant human PSMB5 protein was custom-made by MedChemExpress. Recombinant human ISG20L2 protein was custom-made by Merry Bio Technology Co., Ltd.

Western blot antibodies against PSMB5 (catalog sc-393931), PSMD8 (catalog sc-514053), PSMD3 (catalog sc-393588), PSMC5 (catalog sc-390631), and eIF2a (catalog sc-133132) were purchased from Santa Cruz Biotechnology. Western blot antibodies against ISG20L2 (catalog 24639-1-AP) and GAPDH (catalog 60004-1-Ig) were purchased from Proteintech. Antibodies against ATF4 (catalog 11815), ATF6 (catalog 65880), cleaved caspase-3 (catalog 9661), p-p38MAPK (T180/Y182) (catalog 9211), p38MAPK (catalog 9212), p-eIF2α (S51) (catalog 3398), and XBP-1s (catalog 27901) were obtained from Cell Signaling Technology. Western blot antibodies against p-PERK (T982) (catalog WL05295), PERK (catalog WL03378), and CHOP (catalog WL00880) were obtained from Wanleibio.

### Cell culture.

The human MM cell lines ARD, KMS-11, AMO1, and IM-9 were provided by Qing Yi, Houston Methodist Hospital, Houston, Texas, USA. The human MM cell line OCI-My5 was provided by Jumei Shi, Tongji University School of Medicine, Shanghai, China. All of the above cell lines were cultured in RPMI-1640 medium (HyClone, catalog SH30809.01) supplemented with 10% FBS (GeminiBio, catalog 900-108) at 37°C and 5% CO_2_. All cell lines were authenticated by short tandem repeat profiling and tested for mycoplasma contamination before use.

### Lentivirus packaging and infection.

The pLKO.1 control vector (MilliporeSigma, catalog SHC002) and pLKO.1 plasmids containing human-ISG20L2-shRNAs (sh1: TRCN0000233064 and sh2: TRCN0000233062) were used to produce CTR-KD and ISG-KD viruses, respectively. ISG20L2 cDNA was subcloned into the pLEX-MCS plasmid (Thermo Fisher Scientific, catalog OHS4735) to construct the ISG-OE vector, while the empty pLEX-MCS plasmid was used as a CTR. Lentiviral particles were generated by transfection of HEK293T cells (ATCC) by calcium phosphate precipitation as described earlier (27).

### RT-qPCR.

Total RNA was extracted from cells with TRI reagent (MRC, catalog TR118). cDNA was synthesized by HiScript II Q RT SuperMix (Vazyme, catalog R223-01) following the manufacturer’s protocol. The expression of target genes was analyzed by RT-qPCR using SYBR Green real-time PCR Master Mix (Bimake, catalog B21202, Shanghai, China). The following primers were used for RT-qPCR: GAPDH: Forward ACAACTTTGGTATCGTGGAAGG. Reverse GCCATCACGCCACAGTTTC. ISG20L2: Forward GAGACTCCTACGGTCGATGG. Reverse GGTTGGGTGGCTATTGATCTTTG.

### Cell proliferation assay.

Cell Counting Kit-8 (CCK8) (4A Biotech, catalog AS-20739) was used to detect cellular proliferation. Cells (2 × 10^3^ cells per well) were seeded in 96-well plates in triplicate and examined at 0, 24, 48, and 72 hours after seeding. Cells were incubated with CCK8 solution for 2 hours, and absorbance was measured at 450 nm by a microplate luminometer (Molecular Devices, SpectraMax 190).

### Apoptosis assay.

MM cells were treated with different agents and stained with Annexin V-Alexa Fluor 647/PI Kit (4A Biotech, catalog FXP023). Stained cells were examined by flow cytometry (Beckman Coulter, Navios EX).

### FISH.

FISH assays were performed as described earlier (28). The 1q23 probe, which covered the ISG20L2 gene, was labeled with green fluorescence dye (Anbiping Group). The 1q21 probe was labeled with red fluorescence dye (Anbiping Group). The probe information is summarized in Supplemental Table 1. The results were visualized by a BX51 fluorescence microscope (Olympus), and the images were captured by a FISH imaging system (CytoVision, Leica Biosystems).

### Animal study.

The animal study was approved by the West China Hospital Animal Ethics Committee. A human MM xenograft mouse model was established as previously described (29). CTR-KD or ISG-KD (sh1 sequence was used) ARD cells were intravenously injected (1 million cells per mouse) into 7-week-old, female, severely immunodeficient NOD-*Prkdc^scid^* *IL2rg^tm1^*/Bcgen mice (Biocytogen), 10 mice per group. On day 14 after tumor cell inoculation, the mice injected with CTR-KD or ISG-KD cells were randomly divided into 2 groups (vehicle control and BTZ treatment group, 5 mice per group). The treatment groups were intraperitoneally injected with a low dose of BTZ (0.75 mg/kg, twice a week) for 4 weeks, while the control groups were injected with PBS. The mice were subjected to weekly bioluminescence imaging (IVIS Spectrum, PerkinElmer) to monitor the tumor burden. Mouse serum was collected for ARD monoclonal protein examination using ELISA for human κ light chain (Abcam, catalog ab157709). The mice were sacrificed when they reached preestablished endpoints: paraplegia, lethargy, or body weight loss of more than 20%. The OS of the mice was recorded and compared.

### Proteasome activity assay.

Proteasome-Glo Chymotrypsin-Like Cell-Based Assay (Promega, catalog G8662) was used to determine the intracellular proteasome activity.

### Biotin pull-down assay.

BTZ-b was synthesized and purified with the protocol undisclosed. Streptavidin-coated magnetic beads were obtained from Thermo Fisher Scientific (Dynabeads MyOne Streptavidin T1, catalog 65602). The BTZ-b pull-down assay was performed using a standard protocol. In brief, Dynabead magnetic beads (100 μL per sample pull-down) were washed twice with PBS and incubated with BTZ-b (1 mM, 2 μL) in PBS at 4°C for 2 hours with gentle rotation. Then, the beads were washed twice with ice-cold PBS, resuspended in cell lysate (1 mg/mL, 500 μL PBS) or recombinant protein (1 μg/mL, 500 μL PBS), and incubated at 4°C overnight with gentle rotation. Subsequently, the magnetic beads were washed 5 times with ice-cold PBS, and bound protein was released by adding SDS-PAGE loading buffer. Biotin pull-down served as a negative control.

### SPR assay.

The binding behavior of BTZ or CFZ with ISG20L2 or PSMB5 was measured using SPR on a Biacore X100 system (GE Healthcare, now Cytiva). rISG20L2 or rPSMB5 was immobilized on a CM5 chip through its amine groups. Successful immobilization of ISG20L2 or PSMB5 was confirmed by an approximately 13,000 resonance unit (RU) or an approximately 10,000 RU increase in the sensor chip, respectively. After immobilization, BTZ or CFZ was diluted in running buffer (1× PBS, pH 7.4, 0.05% surfactant P20) at the indicated concentrations and injected at 30 μL/min for 2 minutes. Following analyte injection, running buffer flowed through the sensor surface for a 3-minute period for dissociation. The response was determined as a function of time. *K_D_* values were calculated with Biacore X100 Evaluation software, Version 2.0.2.

### Statistics.

Statistical analyses were performed in GraphPad Prism 8.0.2 software. Significance between 2 groups was determined by 2-tailed Student’s *t* test. One-way ANOVA was performed to estimate differences among 3 or more groups. Two-way ANOVA was performed to compare the differences in the peripheral blood light chain of mice in different treatment groups over time. Patient survival and tumor-bearing mouse survival were analyzed by the log-rank (Mantel-Cox) test. The results are presented as the mean ± standard deviation. *P* ≤ 0.05 was considered statistically significant.

### Study approval.

The use of human bone marrow samples was approved by the Ethical Committee of West China Hospital, Sichuan University (Protocol No. 114). Written informed consent was obtained from the patients or their legal guardians for sample collection and usage. The animal study was approved by the West China Hospital Animal Ethics Committee.

## Author contributions

YY, YG, and JH performed most of the experiments; YZ initiated the project and designed the studies; YZ and JH wrote the manuscript; ZY synthesized BTZ-b; HL, FW, JX, YC, HD, ZL, YQ, LZ, TN, XZ, LY, and TL performed the experiments and provided critical suggestions.

## Supplementary Material

Supplemental data

## Version 1 08/30/2022

In-Press Preview

## Version 2 10/10/2022

Electronic publication
