# Supplementary material for: ISG20L2 suppresses bortezomib antimyeloma activity by attenuating bortezomib binding to PSMB5
Source: JCI Insight. 2022 Oct 10;7(19):e157081. doi: 10.1172/jci.insight.157081 (PMC9675441; doi:10.1172/jci.insight.157081)
Supplement: Supplemental data [file jciinsight-7-157081-s080.pdf]

Supplementary Materials

ISG20L2 Suppresses Bortezomib Anti-Myeloma Activity by Attenuating Bortezomib Binding to PSMB5

Yan Yang, Yuhan Gao, Jingcao Huang, Zhuang Yang, Hongmei Luo, Fangfang Wang, Juan Xu, Yushan Cui, Hong Ding, Zhimei Lin, Xinyu Zhai, Ying Qu, Li Zhang, Ting Liu, Lingqun Ye, Ting Niu, Yuhuan Zheng

Supplementary Table 1. Information of FISH probes

information	1q21 probe	1q23 probe
Upstream STS Maker	STSG627357	D1Z10
Left Primer	TTTCCTTTTGGCTCCTTGTG	GATGGTGCGGTGGTTGAT
Right Primer	CGTCGGACACACCATTGTAG	ACAGGAAAAAAGAGCCAGCA
Downstream STS Maker	SHGC-142645	RH64623
Left Primer	ACATCTGTGGGAGTCAGTGGTTT	TTAGCGGGAGGTGAGAGG
Right Primer	ATGGGCTGTGTTAGCAGTGATTT	TGGGGGAGAGTCATCTGG

Supplementary Figures

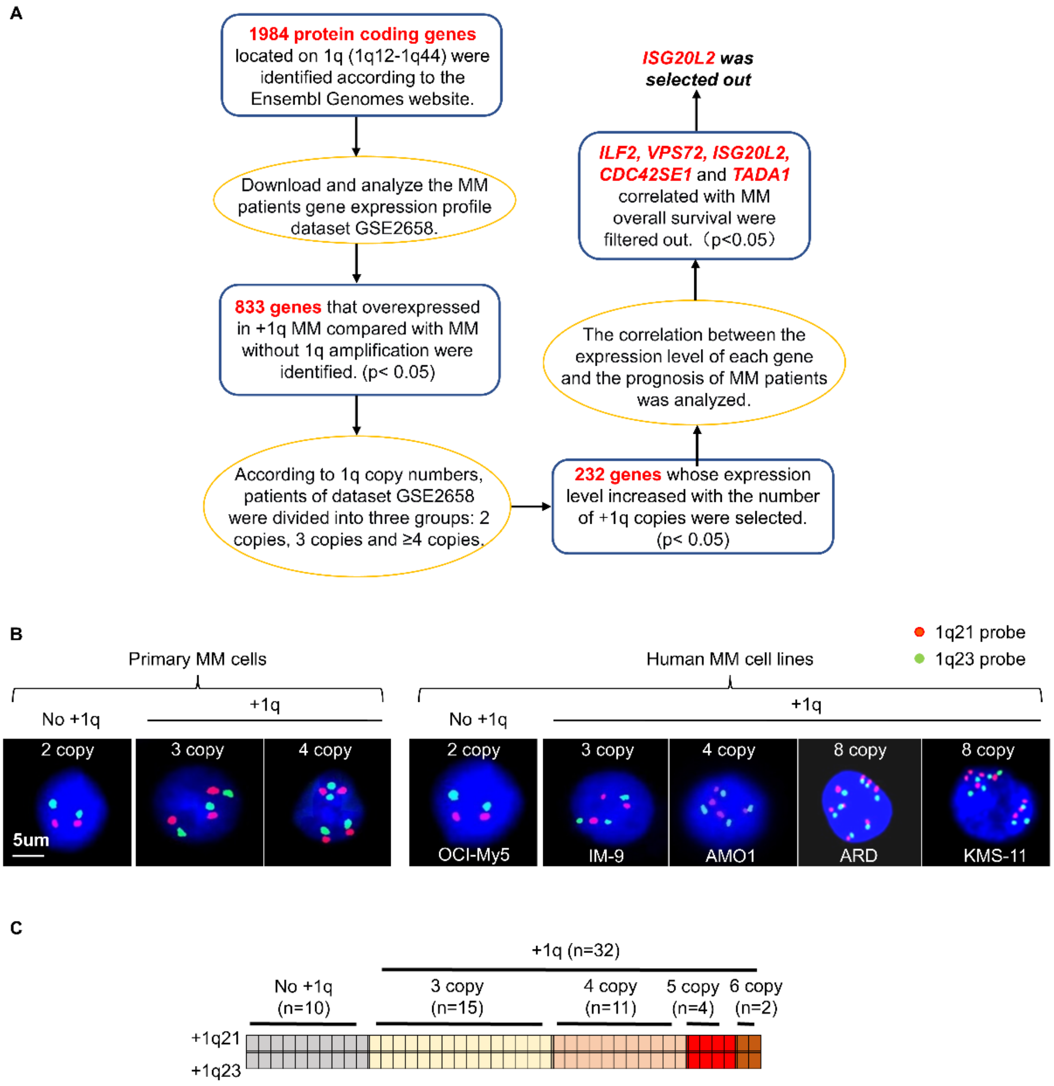

Supplementary Figure 1

A) Schematic algorithm of MM prognostic 1q gene screening. According to the Ensembl Genomes website (https://ensemblgenomes.org/), a total of 1984 protein-coding genes were located on 1q (1q12-1q44). Using the gene expression profile dataset GSE2658 of MM patients, we found 232 genes that were overexpressed as the number of copies of +1q increased ($p < 0.05$). Among those genes, 5 genes correlated with MM overall survival. *ISG20L2* was one identified gene. **B)** Fluorescent in situ hybridization

(FISH) assays using dual-color probes (1q21-red and 1q23-green) were performed in primary MM cells and 5 human MM cell lines. Red (1q21) and green (1q23) probe had identical copy number in the tested samples. **C)** Summarization of FISH assays using primary MM cells of 42 patients (32 cases with 1q amplification and 10 cases without 1q amplification). The same color between upper and lower rows indicated the consistency of 1q21 and 1q23 amplification status.

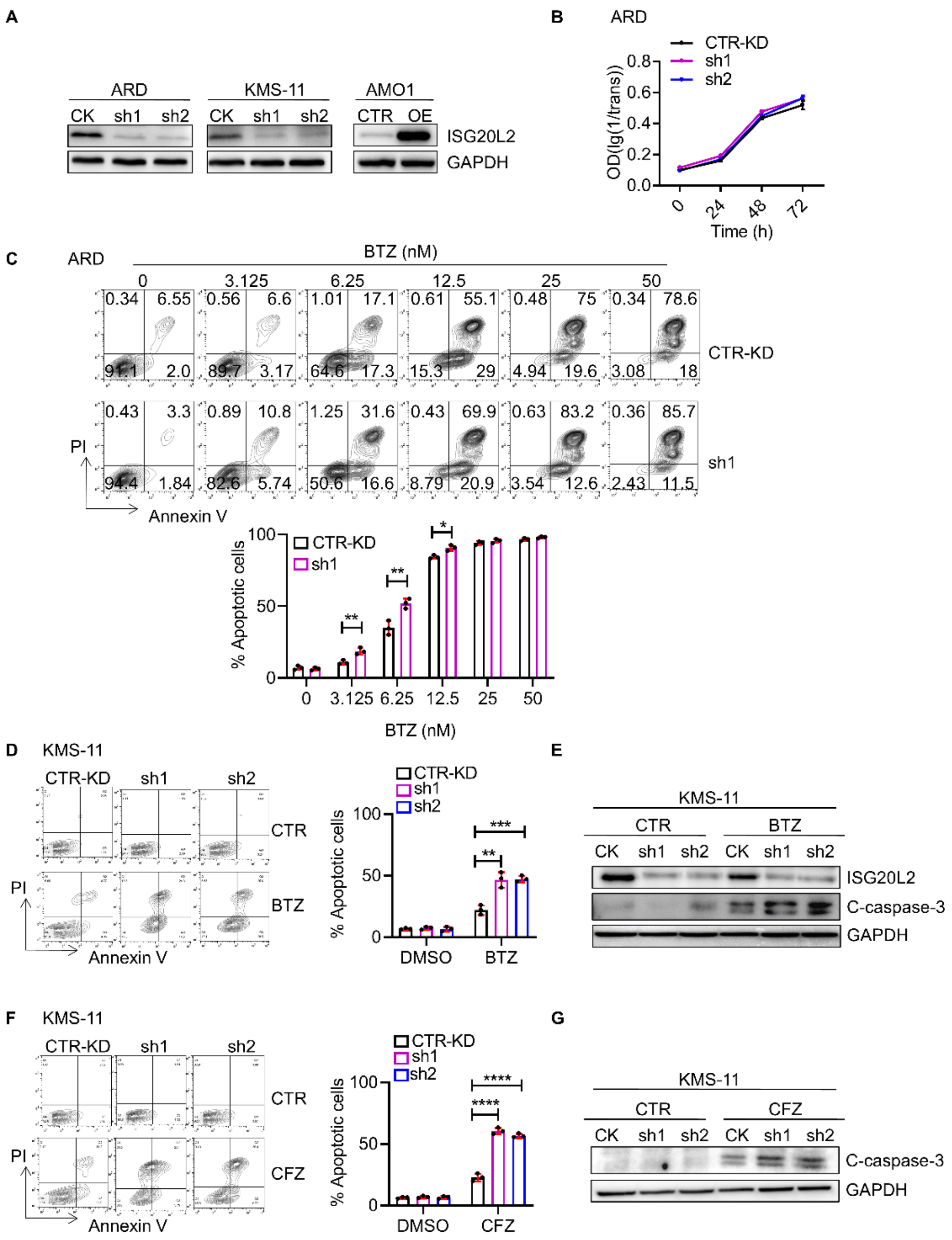

Supplementary Figure 2

A) Western blot showed the decreased expression of ISG20L2 of ARD and KMS-11, which stably expressed *ISG20L2* shRNAs (sh1 and sh2), a non-mammalian target shRNA sequence used as control (CK). AMO1 cell over-expressed ISG20L2, an empty vector used as control (CTR). **B)** ARD cell proliferation of CTR-KD vs. ISG-KD were examined by CCK-8 assay. **C)** Flow cytometry analysis of Annexin V/PI determined

the BTZ (3.125 to 50 nM, 24 h) induced cell death in CTR-KD vs. ISG-KD (sh1 used) ARD cells. Representative flowcytometry data was shown in the upper figure. Lower panel showed the percentage of cell apoptosis at different BTZ doses (n=3). Student's t-test was performed. *P* values are shown as follows: **P*≤0.05; ***P*≤0.01. **D)** Flow cytometry analysis of Annexin V/PI determined BTZ (5nM, 24h) induced cell death in CTR-KD vs. ISG-KD KMS-11 cells. Representative flowcytometry data was shown in the left figure. Right panel showed the percentage of cell apoptosis induced by BTZ (n=3). Student's t-test was performed. *P* values are shown as follows: ***P*≤0.01; *** *P*≤0.001. **E)** Western blot indicated caspase-3 fragmentation in KMS-11 cells treated with 5nM BTZ for 12h. **F)** Flow cytometry analysis of Annexin V/PI determined CFZ (3.5 nM, 24 h) induced cell death in CTR-KD vs. ISG-KD KMS-11 cells. Representative flowcytometry data was shown in the left figure. Right panel showed the percentage of cell apoptosis induced by CFZ (n=3). Student's t-test was performed. *P* values are shown as **** *P*≤0.0001. **G)** Western blot indicated caspase-3 fragmentation in KMS-11 cells treated with 3.5 nM CFZ for 12 h.

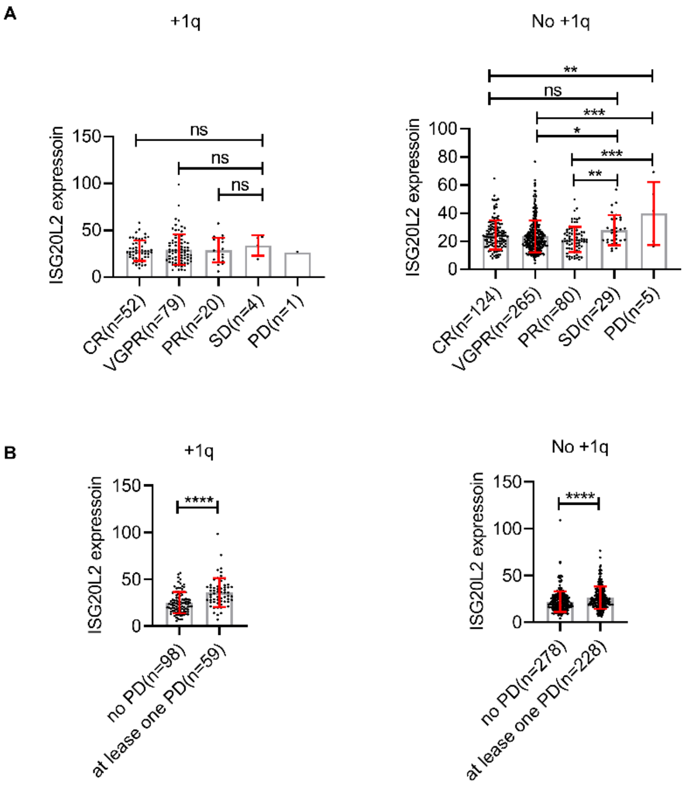

Supplementary Figure 3

A) In MMRF CoMMpass dataset, analyses of the correlation between *ISG20L2* expression and patients' response status to PIs treatment. Patients with 1q amplification (left) were grouped as CR (n=52, *ISG20L2* expression: 8.989 to 57.55, median value=26.80), VGPR (n=79, *ISG20L2* expression: 6.830 to 98.31, median value=26.16), PR (n=20, *ISG20L2* expression: 5.433 to 56.73, median value =26.93), SD (n=4, *ISG20L2* expression: 18.51 to 42.2, median value=37.29) and PD (n=1, *ISG20L2* expression=26.45). Patients without 1q amplification (right) were grouped as CR (n=124, *ISG20L2* expression: 7.612 to 64.42, median value=23.23), VGPR (n=265, *ISG20L2* expression: 4.044 to 76.41, median value=21.38), PR (n=80, *ISG20L2* expression: 6.583 to 49.49, median value =20.75), SD (n=29, *ISG20L2* expression: 12.84 to 56.51, median value=26.39) and PD (n=5, *ISG20L2* expression: 16.14 to 68.98,

median value=41.28). One-Way ANOVA with post hoc LSD-t test was performed. *P* values are shown as follows: ns=*P*>0.05; **P*≤0.05; ***P*≤0.01; *** *P*≤0.001. **B)** In MMRF CoMMpass dataset, analysis of *ISG20L2* expression in PI-treated patients without PD or with at least one PD. Patients with 1q amplification (left) were divided into without PD (n=98, *ISG20L2* expression: 5.433 to 56.73, median value=23.12) and with at least one PD (n=59, *ISG20L2* expression: 6.83 to 98.31, median value=34.85). Patients without 1q amplification (right) were divided into without PD (n=278, *ISG20L2* expression: 4.044 to 108.9, median value=20.24) and with at least one PD (n=228, *ISG20L2* expression: 6.284 to 76.41, median value=23.79). Student's t-test was performed. *P* values are shown as **** *P*≤0.0001.

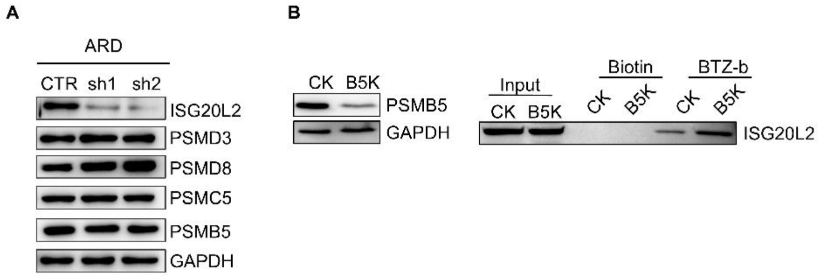

Supplementary Figure 4

A) Western blot results showed the expression of proteasome subunits in CTR-KD vs. ISG-KD ARD cells. **B)** Western blot showed the knockdown of PSMB5 (BK5) expression in ARD cell (left figure). Biotinylated-BTZ pull-down assay using CTR-KD and PSMB5 knockdown ARD cell lysate (right figure).

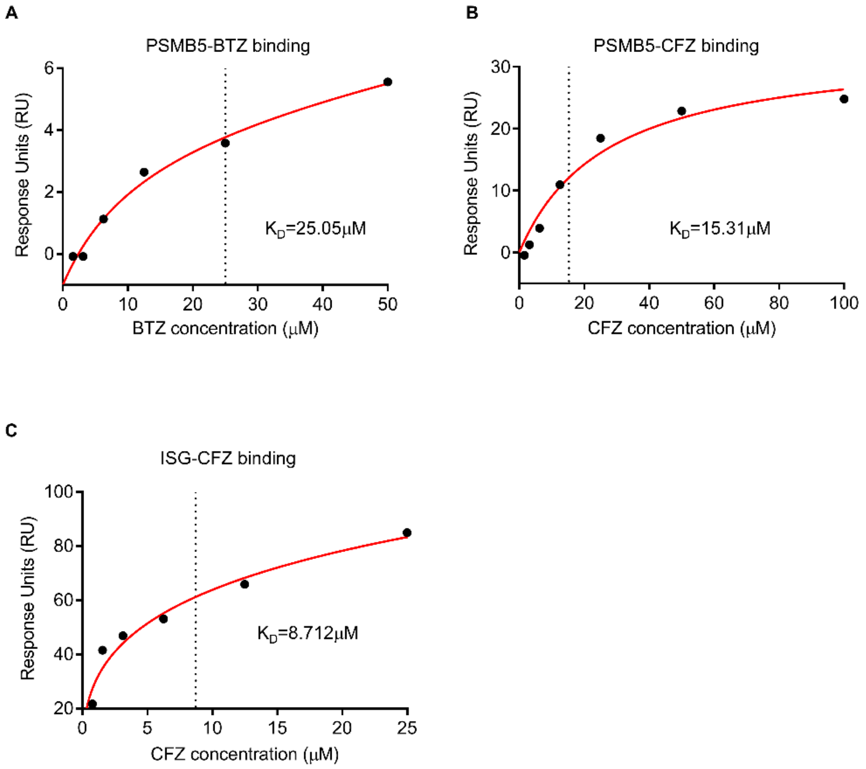

Supplementary Figure 5

A) Graphs of equilibrium RU responses of BTZ binds to PSMB5 measured by SPR versus compound concentrations were plotted. The estimated K_D is 25.05 μM. **B)** Graphs of equilibrium RU responses of CFZ binds to PSMB5 measured by SPR versus compound concentrations were plotted. The estimated K_D is 15.31 μM. **C)** Graphs of equilibrium RU responses of CFZ binds to ISG20L2 measured by SPR versus compound concentrations were plotted. The estimated K_D is 8.712 μM.
